# New Myzostomids (Annelida) in Symbiosis with Feather Stars in the Shallow Waters of the South China Sea (Hainan Island)

**DOI:** 10.3390/ani14152265

**Published:** 2024-08-04

**Authors:** Alexander Isaychev, Dimitry Schepetov, Yutong Zhou, Temir A. Britayev, Viatcheslav N. Ivanenko

**Affiliations:** 1 Biological Faculty, Shenzhen MSU-BIT University, Shenzhen 518172, China; isaychev1@yandex.ru (A.I.); d.m.schepetov@gmail.com (D.S.); 1120210251@smbu.edu.cn (Y.Z.); 2 Department of Invertebrate Zoology, Lomonosov Moscow State University, Moscow 119992, Russia; 3 A.N. Severtsov Institute of Ecology and Evolution Russian Academy of Sciences, Moscow 129164, Russia; britayev@yandex.ru

**Keywords:** Myzostomida, annelids, feather stars, Crinoidea, Comatulidae, Hainan Island, Sanya, South China Sea, marine biodiversity, symbionts, feather stars

## Abstract

**Simple Summary:**

Simple Summary: This research details the discovery of three new myzostomid worm species from feather stars near Sanya, Hainan Island. Additionally, we report the presence of *Myzostoma polycyclus* in the South China Sea for the first time. These findings, combined with an analysis of available molecular data, underscore significant gaps in our understanding of marine biodiversity of myzostomids.

**Abstract:**

This research delves into the molecular and morphological characteristics of myzostomid worms associated with common shallow-water feather stars (Echinodermata: Crinoidea: Comatulidae) in the coastal waters near Sanya, Hainan Island. Through the examination of specimens collected at depths of up to 10 m using scuba diving techniques, we describe three new species (*Myzostoma ordinatum* sp. nov., *M. scopus* sp. nov., and *M. solare* sp. nov.) and report the first record of *Myzostoma polycyclus* Atkins, 1927 in the South China Sea. The absence of overlap with the seven previously documented Myzostomida species in the shallow waters of Hong Kong and Shenzhen reveals significant gaps in our understanding of marine biodiversity in the South China Sea. These findings, combined with an analysis of available molecular data, underscore the potential existence of unexplored and diverse symbiotic relationships among marine invertebrates within the region.

## 1. Introduction

The Myzostomida Graff, 1877, represents a distinctive group of diminutive and highly specialized symbiotic worms primarily associated with crinoids. They exhibit a unique body plan that markedly diverges from other Annelida taxa, with their specialized adaptations highlighted by Eeckhaut and Lanterbecq [1]. These worms are globally distributed, predominantly found in subtidal tropical zones, known as symbionts of diverse feather stars [2]. The morphological diversity within the Myzostomida group is linked to their symbiotic associations with echinoderm hosts, facilitating a multitude of body shapes. This morphological variance reflects their assorted ecological niches, ranging from free moving to cysticolous and gallicolous forms, in addition to those inhabiting the digestive systems of their hosts as endobionts [3].

Despite their apparent diversity, the taxonomic representation of Myzostomida remains underrepresented in the scientific literature, with a substantial number of undescribed species as emphasized by Summers, Al-Hakim, and Rouse [4]. Presently, approximately 180 species of Myzostomida, classified into seven families, are known, with 159 species attributed to the genus *Myzostoma* Leuckart 1836 [5,6,7]. The majority of myzostomids are ectocommensal on comatulids, although some inhabit the host’s body, forming cysts, galls, or residing within the digestive system [8]. Myzostomids are also found to be associated with Asteroidea, Ophiuroidea [9], and Antipatharia [10], and they are reported to inhabit diverse oceanic regions, from subtidal zones to depths exceeding 3000 m.

Hainan Island exhibits significant temporal and spatial variability in its shoreline and shallow-water community structures, resulting in marked fluctuations in species composition and population densities [11]. It is recognized as a biodiversity hotspot within the South China Sea, positioned at the interface between the China–Japan Subtropical Subregion and the Indo-Malaysian Tropical Subregion. Hainan Island represents the southernmost reach of the cold current’s surface flow along Guangdong province’s coastline [12]. Despite its ecological significance, there is a lack of documented records on Myzostomida diversity in this region.

This study is part of a broader survey investigating the symbiotic fauna of macroinvertebrates within tropical coral communities [13,14,15,16,17,18,19,20,21,22]. It focuses on the integrative taxonomy of Myzostomida worms associated with common shallow-water feather stars of Hainan Island.

## 2. Materials and Methods

### 2.1. Sample Collection and Processing

**Figure 1:**
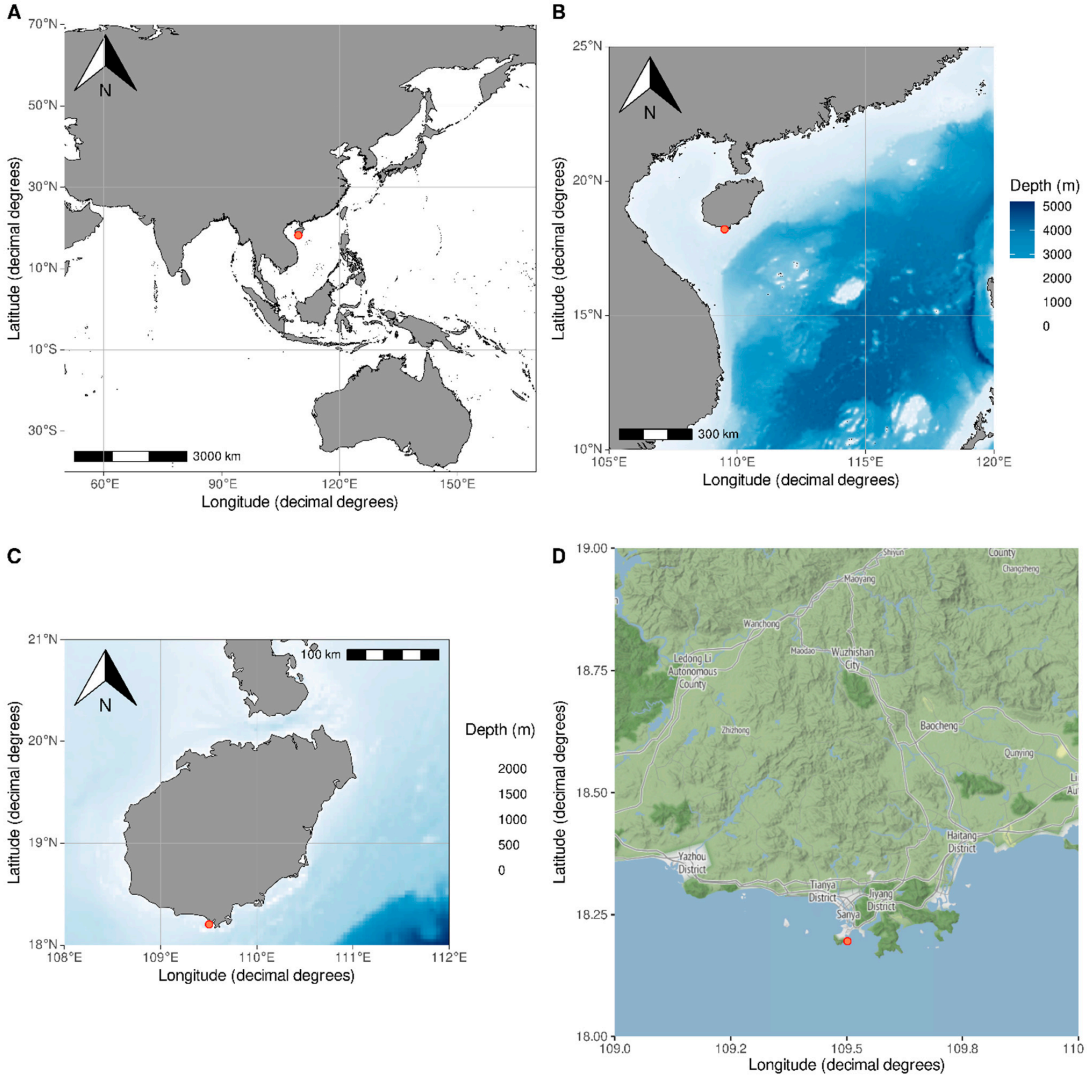
Sampling locality and sampling site (red circle). (**A**–**D**)—Regional view showcasing the geographic area around the sampling site. (**B**,**C**)—Detailed sampling site enhanced with bathymetric data, highlighting underwater topography and depth details.

Symbionts (copepods, shrimps and worms) of two common shallow-water feather stars, *Comanthus parvicirrus* (Müller, 1841) and *Comaster schlegelii* (Carpenter, 1881) (Comatulidae, Comatulida), were collected using a local boat and scuba diving in the coastal waters near Sanya City, Hainan Island (N 18°12.279′ E 109°30.197′) (Figure 1).

The feather stars were collected at depths of 5 to 8 m and placed in resealable plastic bags underwater. Upon retrieval to the surface, a 10% ethanol solution was added to the bags. After 30 min of waiting and gentle shaking, the liquid was passed through a sieve with a 100-micrometer mesh diameter. The residue was preserved in 95% ethanol and stored at a temperature of −20 °C. The maps were generated using the ‘ggplot2’ package in R, version 2.1.1 [23].

This examination of six samples revealed 116 myzostomid specimens (see Table 1). The chosen preservation method was instrumental in maintaining the structural integrity of the specimens, facilitating their use in future morphological and molecular investigations after long storage.

All examined materials are in the collections of the Zoological Museum of Moscow State University, Russia (ZMMU), and the Faculty of Biology at MSU-BIT Shenzhen University, China. The museum specimen voucher numbers and NCBI GenBank Accession Numbers for our samples are in Table 2 and Table S1.

### 2.2. Morphological Analysis 

For morphological observation, a dissecting stereomicroscope Cnoptec SZ650 (Chongqing, China) was used. Each collected specimen underwent a comprehensive external morphological examination, encompassing a thorough assessment of various attributes such as patterns of coloration, body morphology, surface texture, and appendage characteristics. This detailed examination and measurements were carried out under the scrutiny of a compound microscope Olympus BX43FC (Tokyo, Japan). 

Species categorization and identification were executed with a reliance on the most up-to-date published descriptions of myzostomid species. From each of the identified species, a judicious selection process was undertaken to choose specimens with the most pristine and intact external morphological features. These selected specimens were subjected to a detailed photographic documentation process, utilizing a high-resolution imaging stereomicroscope. Additionally, images were captured using a scanning electron microscope (SEM) KYKY EM6200 (Beijing, China). 

The SEM specimen preparation procedure involved a protocol that included the drying of specimens within a Labconco FreeZone 2.5 Liter -84C Benchtop Freeze Dryer (Kansas City, MO, USA) maintained at a temperature of −50 °C and a pressure of 0.03 mbar for a duration of four hours. Subsequently, a one-minute gold coating was applied to the specimens using a vacuum sputter.

### 2.3. Molecular Analysis

For brief molecular species identity analysis, we used a shortened most informative fragment of mitochondrial cytochrome c oxidase subunit I (COI) gene. DNA was extracted on magnetic beads using OMEGA Bio-Tek E.Z.N.A. Mag-Bind Blood & Tissue DNA HDQ 96 kit (Norcross, GA, USA), according to manufacturer’s protocol. Target COI fragment was amplified using Sauron-S878 [24] and jgHCO2198 [25] with 5′v M13 [26] sequencing adapters (M13F_Sauron-S878 tgtaaaacgacggccagtGGDRCWGGWTGAACWGTWTAYCCNCC; M13R_jgHCO2198 caggaaacagctatgacTAIACYTCIGGRTGICCRAARAAYCA) primer pair. Reactions were performed using Clontech TaKaRa cellartis Premium Taq PCR kit (Kyoto, Japan). Amplicones were sequenced in both directions with M13F(-21) (TGTAAAACGACGGCCAGT) and M13R(-27) (CAGGAAACAGCTATGAC) sequencing primers [26] at Sangon Biotech (Shanghai, China).

Consensus sequences for each sample were assembled from raw reads in both directions and checked for ambiguities and low-quality base identifications in Geneious R10 [27]. Combined sequences were checked for putative contamination by using BLAST-n algorithm at GenBank nr/nt database [28]. Original data and publicly available sequences for genus *Myzostoma* were aligned with the MUSCLE [29] algorithm implemented in MEGA 7.0 [30]. Other Myzostomids were used as outgroups. Additionally, all sequences were translated into amino acids to verify reading frames and check for stop-codons and to avoid pseudo-gene sequence contamination. A total of 125 sequences were used, including outgroups; resulting alignment length was 622 bp (Table S1). 

Bayesian phylogenetic reconstruction of phylogeny (BI) was performed in MrBayes 3.2 [31]. Markov chains were sampled at intervals of 500 generations. Analysis was initiated with random starting tree and ran for 5 × 10^7^ generations. Maximum likelihood phylogeny inference (ML) was performed in the HPC-PTHREADS version of RaxML 8.2.12 [32], with 400 pseudo-replicates of fast bootstrap under the GTRCAT model of nucleotide evolution. Number of sufficient bootstrap pseudo-replicates was determined using AutoMRE approach [33]. Final phylogenetic tree images were rendered in FigTree 1.4.0 and further modified in Inkscape 1.1. Attribution to marine biogeographic realms followed borders from Costello et al. [34] and used ranges described in Summers et al. [4] and data from original species descriptions.

## 3. Results

The myzostomids associated with the three specimens of *Comanthus parvicirrus* comprised a single species, *Myzostoma polycyclus* Atkins, 1927. Among the myzostomid specimens collected from the four specimens of *Comaster schlegeli* (Carpenter, 1881), three new species were identified: *Myzostoma ordinatum* sp. nov., *Myzostoma scopus* sp. nov., and *Myzostoma solare* sp. nov.

### 3.1. Taxonomy

*Myzostoma scopus* sp. nov. Isaychev, Britayev, Ivanenko

Figure 2A–D

http://zoobank.org/41B6E07C-6799-425B-9706-76F756BA1E97

*Holotype*: (ZMMU Pl-4886) One specimen in 96% ethanol. Sampling site is in the vicinity of Sanya city, Hainan Island (N 18°12.279′ E 109°30.197′), depth 5–8 m. Collected using scuba on 25 November 2009 by T.A. Britayev and V.N. Ivanenko. 

*Remaining specimens*: Two specimens in 96% ethanol. Vicinity of Sanya city, Hainan Island (N 18°12,279′ E 109°30′197), depth 5–8 m. Collected on 25 November 2009 by T.A. Britayev and V.N. Ivanenko.

*Hosts and distribution*: *Comaster schlegelii* (Carpenter, 1881) depth 5–8 m, South China Sea, Hainan Is., Sanya.

*Etymology:* The name “scopus” is derived from the Latin word “scopus”, meaning “target”, and alludes to its distinctive pigmentation pattern, which resembles an archery target.

**Figure 2:**
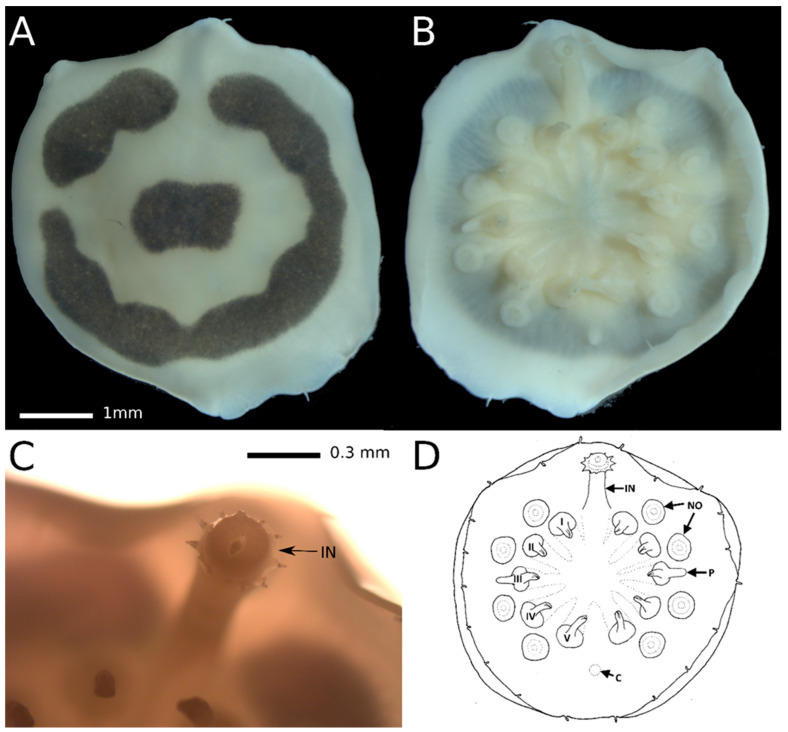
*Myzostoma scopus* sp. nov., holotype. (**A**)—habitus, dorsal view, (**B**)—habitus, ventral view, (**C**)—introvert arrowed, ventral view, (**D**)—habitus, ventral side outline. Abbreviations: IN—introvert, NO—lateral organ, P—penis, C—cloaca.

*Description and diagnosis*: The body of the holotype is circular, approximately 5 mm in diameter. The dorsal surface exhibits a smooth texture and is of ivory color, featuring a black laterally elongated rectangular spot at its center, surrounded by an interrupted ring of the same black color as the central spot (Figure 2A). The two other specimens from another host demonstrate similar color pattern, but without a central spot and less contrast than in the holotype (dark brownish-pink ring on light brownish-pink surface). The ventral surface is uniformly ivory in color in the holotype (Figure 2B,D) and uniformly light brown pinkish in the other two specimens. The centers of parapodial bases are positioned approximately 40% along the path from the center of the ventral side to the body margin. The parapodia are around 0.6–0.7 mm long and consist of two parts of roughly equal length: a stout conical base with a diameter of about 0.35 mm in its proximal part and a less massive cylindrical distal part with a rounded tip. Each parapodium is equipped with a hook. Parapodia are evenly distributed around the circular body. In our specimen, the parapodia bend toward the center, revealing the absence of median cirri. The outer side of the third parapodia carries a penis, measuring approximately 0.4 mm in length and exhibiting a similar shape to the distal part of the parapodium. Prominently visible large (approximately 0.3 mm) lateral organs, situated at about 30% along the path from the outer side of the parapodia to the body margin, alternate between the parapodia. The everted introvert (Figure 2C) is 3.5 times as long as it is wide and bears ten acute papillae. The cloaca is positioned at the same distance from the body center and margin as the lateral organs. The body margin is smooth, thin, and semitransparent, hosting 20 very short (0.15–0.18 mm) cirri of the same digitiform shape. The extreme edge of the body margin slightly curves on the ventral side, possibly due to ethanol fixation. The cirri are evenly distributed around the body margin.

*Remarks: Myzostoma scopus* sp. nov. displays several distinguishing characteristics, although there are a few myzostomid species that bear some resemblance to it, particularly in terms of having a circular body equipped with 20 small finger-like cirri around its margin. A detailed morphological comparison is provided below.

*Myzostoma scopus* sp. nov. is closely related morphologically and genetically to *M. susanae* Summers and Rouse in Summers, Al-Hakim, and Rouse, 2014, originally described from *Comaster schlegelii* (Carpenter, 1881) found at a depth of 6 m at Lizard Island Reef, Australia (Figure 2). However, unlike *M. susanae*, it exhibits a distinctive pigmentation pattern on its dorsal side and possesses more prominent cirri.

Another species that shares similarities is *M. coriacium* Graff, 1884, collected from *Colobometra perspinosa* (Carpenter, 1881) at depths of 7.5–10 m in Port Denison, Australia. According to Graff’s description, *M. coriacium* is not transparent and appears darkish brown in ethanol. It also bears lateral organs located halfway between the center and margin of the body. Graff’s illustration of *M. coriacium*’s cross-section depicts a body that is deeply ventrally concave and dorsally convex. In contrast, *M. scopus* sp. nov. can be distinguished by its color, a semitransparent body margin that is slightly bent ventrally, and lateral organs positioned closer to the body margin.

*M. scopus* sp. nov. shares a color pattern resembling that of *M. horologium* Graff, 1884, but differs by having a notably shorter parapodia, which do not extend beyond the body margin.

In comparison to *M. brevipes*, another species with a circular body, *M. scopus* sp. nov. can be distinguished by its smooth dorsal side, the absence of a dorsomedial ridge, and the absence of radial folds on the dorsal side.

When compared to *M. seymourcollegiorum* Rouse and Grygier, 2005, found on *Cenolia trichoptera* (Müller, 1846) and *Cenolia glebosus* Rowe, Hoggett, Birtles, and Vail, 1986 in southern Australia, *M. scopus* sp. nov. can be distinguished by the absence of parapodial cirri.

Differentiating from both *M. pallidum* Graff, 1877 (recorded from *Comatula solaris* and *Comanthus parvicirrus*) and *M. triste* Graff, 1877 (recorded from *Comanthus parvicirrus*), both from Bohol strait, *M. scopus* sp. nov. is characterized by its notably shorter parapodia, finger-like cirri with thin bases, and distinct coloration.

*Myzostoma polycyclus* Atkins, 1927

Figure 3A

*Studied material*: 33 specimens from *C. parvicirrus*. 

*Type locality:* Pacific: Torres Strait. Host of the type locality: *Comanthus parvicirrus;* depth unknown [35].

*Other hosts and distribution*: *Comanthus suavius* (Rowe, Hoggett, Birtles, and Vail, 1986), *Capillaster multiradiatus* (Linnaeus, 1758), *Clarkcomanthus littoralis* (Carpenter, 1888), *Comanthus parvicirrus*. North Sulawesi, Papua New Guinea, Fiji, Japan, South China Sea, Hainan Island, Sanya. Depth 2–9 m ([4], our data). 

**Figure 3:**
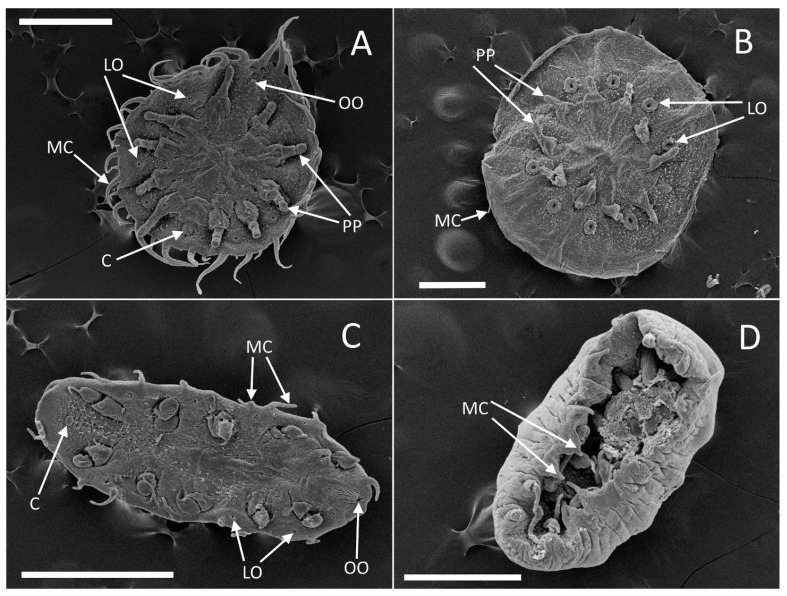
(**A**) *Myzostoma polycyclus,* ventral view (SEM); (**B**) *M. solare* sp. nov., ventral view (SEM); (**C**) *M. ordinatum* sp. nov., ventral view; (**D**) *M. ordinatum* sp. nov., ventral view (SEM). Scale bar = 500 µm. Abbreviations: C—cloaca, LO—lateral organs, MC—marginal cirri, OO—oral opening, PP—parapodia.

*External morphology* (Figure 3A): Examined material conforms to original [35] and consequent descriptions [36,37]. Specimens from various samples exhibited varying colors, which correlated with the coloration of their respective hosts (beige or dark blue). However, no morphological differences were observed among them, consistent with previous findings.

*Remarks:* The wide distribution and diversity of hosts, along with the molecular data obtained by us and deposited in GenBank, indicate on one hand the potential for widespread occurrence and diversity. However, they also highlight challenges in verification and potential inaccuracies in identifications.

*Myzostoma solare* sp. nov. Isaychev, Britayev, Ivanenko

**Figure 4:**
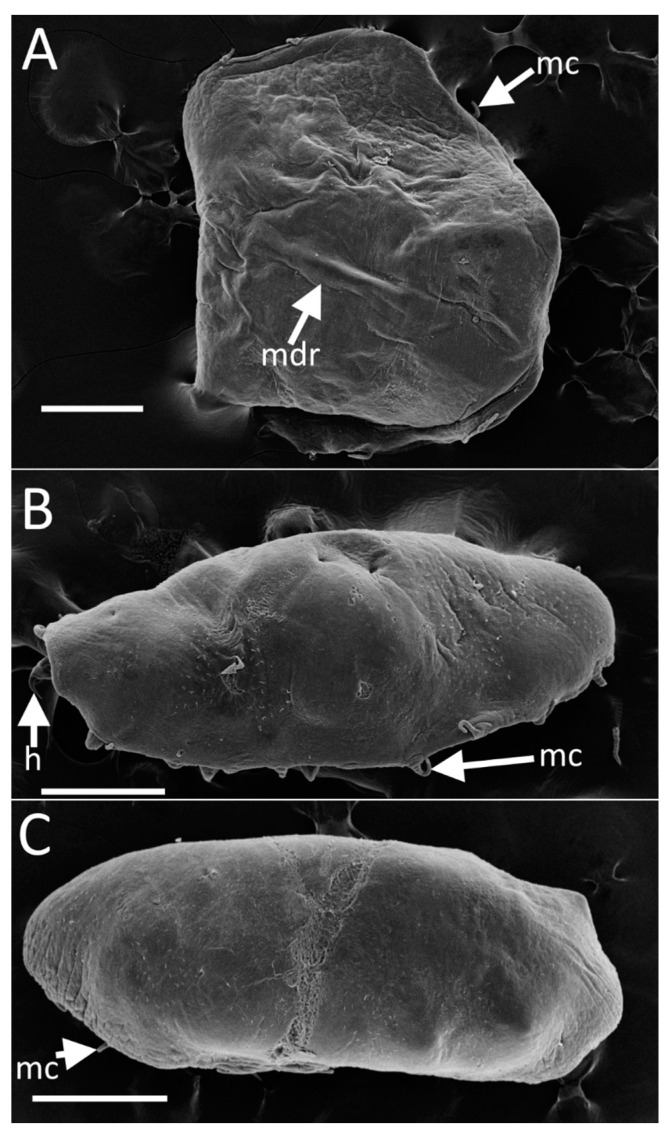
(**A**)—*Myzostoma solare* sp. nov. Dorsal view, SEM. (**B**,**C**)—*Myzostoma ordinatum* sp. nov. dorsal view. Abbreviations: h—hook of parapodium, mc—marginal cirri, mdr—mid-dorsal ridge. Scale bar = 300 µm.

Figure 3B and Figure 4A

http://zoobank.org/41B6E07C-6799-425B-9706-76F756BA1E97

*Holotype*: (ZMMU Pl-4887) One specimen in 96% ethanol. Sanya, Hainan Island (N 18°12.279′ E 109°30.197′), depth 5–8 m. Collected on 25 November 2009 by T.A. Britayev and V.N. Ivanenko. 

*Hosts and distribution*: *Comaster schlegeli* (Carpenter, 1881), depth 5–8 m, South China Sea, Hainan Island, Sanya.

*Etymology:* The species name “solare” is derived from the Latin word “solare”, meaning “solar” in the neutral gender, in reference to its circular shape.

*Studied material*: Eight specimens from *Comaster schlegeli* (Carpenter, 1881). 

*External morphology*: The body is circular (Figure 3B) and flat, measuring 1–1.2 mm in diameter. It exhibits a grayish-beige coloration with a darker region in the center of the dorsal side. The body is rounded evenly and is adorned with twenty prominent finger-like cirri, each measuring 0.08–0.1 mm in length. The dorsal surface displays a median ridge and radial folds (Figure 4A), while the ventral surface features scattered ciliary bundles. Parapodia are positioned at approximately 40% of the distance from the center of the body to the body margin, measuring 0.2 mm in length. Lateral organs are located roughly a quarter of the distance from the parapodial bases to the body margin and have a diameter of about 0.1 mm.

*Remarks*: *Myzostoma solare* sp. nov. exhibits close affinities with *Myzostoma brevipes* Graff, 1884, originally described from *Crinometra brevipinna* (Pourtalès, 1868) (formerly *Antedon pourtalèsi* Carpenter, 1888) in the Caribbean Sea at depths of 300 m. However, *M. brevipes* is distinguishable from the new species by its notably shorter parapodia. Moreover, *Myzostoma solare* sp. nov. also exhibits close affinities with *M. pallidum* Graff, 1877, described from *Comatula solaris* Lamarck, 1816, and *Comanthus parvicirrus* from shallow waters in the Philippines. Nonetheless, *Myzostoma solare* sp. nov. diverge from *M. pallidum* by possessing slender, finger-like marginal cirri, as opposed to the triangular cirri characteristic of *M. pallidum.* Despite their sister-group phylogenetic position, *M. solare* sp. nov. and *M. polycyclus* exhibit pronounced morphological differences. *M. solare* sp. nov. is characterized by about twenty small, uniform marginal cirri, whereas *M. polycyclus* features a body margin adorned with approximately fifty long, robust cirri of two distinct, alternating shapes.

*Myzostoma ordinatum* sp. nov. Isaychev, Britayev, Ivanenko

**Figure 5:**
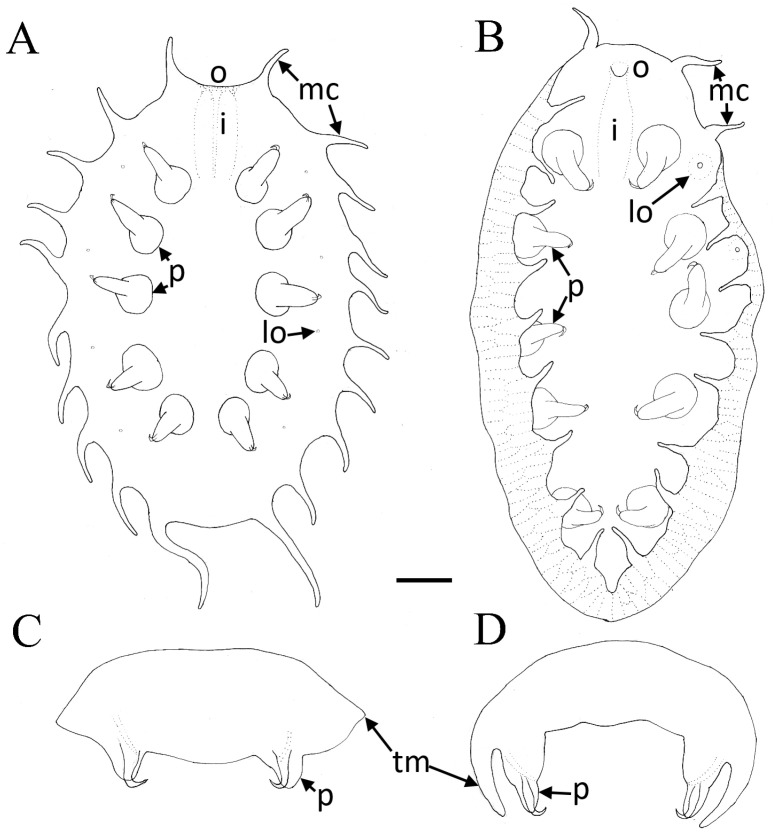
Variability in the morphology of *Myzostoma ordinatum* sp. nov. (**A**,**B**)—Ventral view outlines depicting different shapes. (**C**,**D**)—Ventral view outlines and schematic cross-sections at the level of the third pair of parapodia showing diverse morphologies. Scale bar = 200 µm. Abbreviations: i—inversed introvert; lo—lateral organs; mc—marginal cirri; p—parapodia; tm—trunk margin.

Figure 3C,D, Figure 4B,C, Figure 5A–D and Figure S1

http://zoobank.org/41B6E07C-6799-425B-9706-76F756BA1E97

*Holotype*: (ZMMU Pl-4889) One specimen in 96% ethanol. Sampling site is in the vicinity of Sanya city, Hainan Island (N 18°12.279′ E 109°30.197′), depth 5–8 m. Collected using scuba on 25 November 2009 by T.A. Britayev and V.N. Ivanenko. 

*Hosts and distribution*: *Comaster schlegelii* (Carpenter, 1881), depth 5–8 m, South China Sea, Hainan Island, Sanya.

*Etymology:* The name “*ordinatum*” is derived from the Latin adjective which means “regular” in neutral gender, due to the even situation of cirri around body margin.

*Studied material:* 28 specimens from 3 specimens of *C. nobilis*.

*External morphology*: The body is elongated (Figure 3C,D, Figure 4B,C and Figure 5A,B), measuring between 0.8 and 1.2 mm, and is oval, with a length approximately twice its width and expressed dorsal convexity and ventral concavity. The dorsal surface appears smooth and is colored yellowish gray, occasionally exhibiting a lighter dorsomedial stripe in certain specimens. SEM observation displays the existence of dispersed ciliary bundles on the dorsal side (Figure 4B,C). On the ventral side, parapodia emerge from cavities and can extend to the body margin, sometimes even surpassing the margin, making them visible from a dorsal view. The length of the parapodia is approximately one-third of the maximum body width. There are twenty finger-like cirri in total, with a broad base tapering distally, each measuring about one-fifth the length of the maximum body width, while the posterior-most pair of cirri on some specimens are about half the maximum body width long. Four pairs of lateral organs are situated on low mounds, positioned midway between the base of the parapodia and the body margin. These lateral organs and parapodia alternate in arrangement. The everted introvert is roughly half the body width in length and is adorned with four distal papillae (Figure S1). The oral opening is almost terminal and oriented toward the anterior body margin.

*Remarks.* The specimens of *Myzostoma ordinatum* sp. nov. display certain similarities with *Myzostoma longitergum* Eeckhaut, Grygier, Deheyn, 1998, known from *Comaster schlegelii* and *Comaster audax* in Papua New Guinea waters. However, they lack the characteristic cirri 2–9 grouped in doublets flanking lateral organs as described in the original species account. Moreover, the marginal cirri of *M. ordinatum* sp. nov. are evenly distributed around the body margin, contrasting with the anteriorly shifted arrangement in *M. longitergum.*

The morphological features of *M. ordinatum* sp. nov. resemble that of *M. compressum* Graff, 1884, described from *Bathycrinus aldrichianus* in the subantarctic Indian Ocean at a depth of 2.5 km. Nevertheless, our specimens were discovered in shallow tropical waters on a different host, lacking the distinctive mid-dorsal ridge of *M. compressum* and instead featuring a uniformly rounded dorsal surface. *M. ordinatum* sp. nov. close *M. nasonovi* Fedotov, 1938, documented from Hong Kong, but can be distinguished by twenty larger cirri compared to the small cirri of *M. nasonovi.*

Several specimens of *Myzostoma ordinatum* sp. nov. exhibit a convex dorsal side and concave ventral side, with body margins folded over the ventral surface to varying degrees (Figure 5C,D). In some cases, the folds were so pronounced that they completely obscured the ventral side, potentially influenced by preservation in alcohol. We assume that the specimens may demonstrate a natural ventral concavity and dorsal convexity, possibly facilitating a snug fit within the ambulacral groove of their host feather star.

### 3.2. Molecular Analysis 

**Figure 6:**
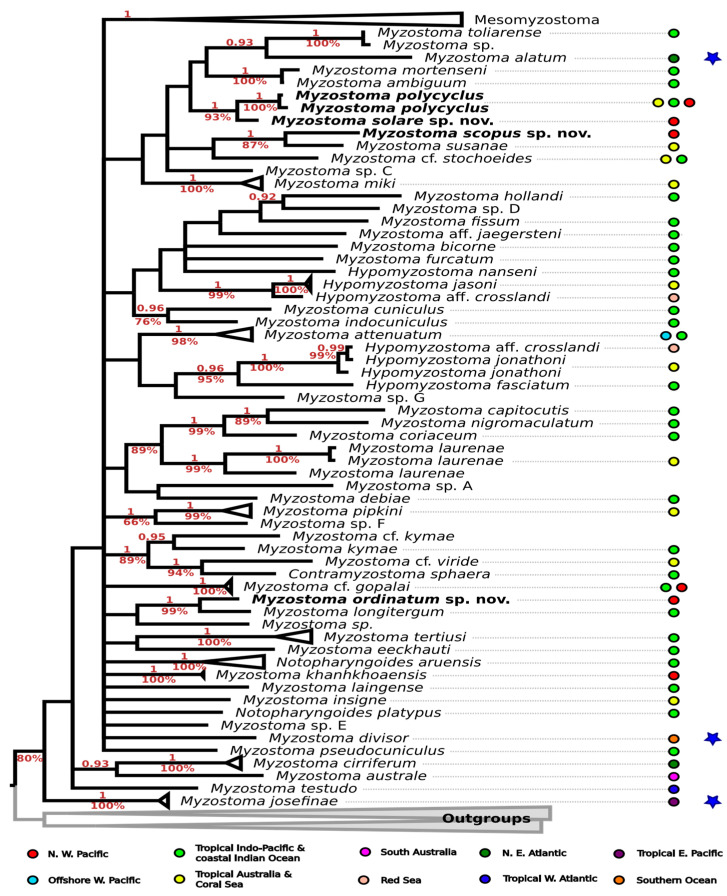
Molecular phylogenetic reconstruction of the genus *Myzostoma* based on partial COI sequences, Bayesian inference. Species-level clades and outgroups are collapsed to a single branch. Numbers above branches indicate posterior probabilities (PP) from Bayesian inference, numbers below branches indicate bootstrap support from maximum likelihood (BS). Only PP > 0.9 and BS > 60 are shown. Geographical ranges (see according to Figure S4) indicated by colored circles and according to color scheme are annotated below the phylogenetic tree; species known from aphotic zone marked by blue star.

We sequenced fragments of the COI gene for four myzostomid species, including those described as new in this study. GenBank accession numbers for these new sequences are provided in Table 2. Phylogenetic analysis using both publicly available and newly generated data for myzostomid COI (Figure 6) reveals unresolved relationships using both reconstruction methods. Detailed phylogenetic trees, along with accession numbers, are provided in the supplementary figures (Figures S2 and S3).

The genus *Myzostoma* does not form a monophyletic group and instead clusters with samples of *Hypomysostoma* and *Mesomyzostoma*, showing moderate support in maximum likelihood reconstructions. This lack of monophyly within *Myzostoma* has been previously observed in studies dedicated to myzostomid phylogeny (e.g., [2]).

*Myzostoma solare* sp. nov. is consistently reconstructed as closely related to our samples of *M. polycyclus* (1PP/100%BS), with robust support in both phylogenetic approaches (1PP/93%BS). *M. polycyclus* (DQ238202) forms another distinct clade (1PP/100%BS) with *M. toliarense* (DQ238201), having nearly identical COI sequences. *M. scopus* sp. nov. is recovered as the sister to *M. susanae*, supported by high Bayesian inference (1PP/87%BS).

Our specimen of *M. ordinatum* sp. nov. forms a well-supported clade (1PP/99%BS) with a publicly available sequence of *M. longitergum* (KM014193). However, the sequences overlap with only 94.7% identical bases, indicating genetic divergence between the two species.

## 4. Discussion

Our study reveals the presence of four species in the northern part of the South China Sea, three of which are new scientific discoveries. Combined with the literature and molecular data available in GenBank, these findings underscore the limited understanding of this intriguing group of diminutive symbiotic invertebrates. In the coastal waters of China, only a small fraction of the global diversity of Myzostomida species (7 out of 180 known species) has been previously documented in the vicinity of Hong Kong and Shenzhen City [38]. These include *Myzostoma attenuatum* (Grygier, 1989), *M. antennatum* (Graff, 1884), *M. bocki* (Jägersten, 1937), *M. dodecaphalcis* (Grygier, 1992), *M. nasonovi* (Fedotov, 1938), *M.* cf. *pallidum* (Graff, 1877), and *M. lobatum* (Graff, 1877). This limited data documentation contrasts with the broader richness of feather stars in the South China sea, where at least 44 species have been reported [39], suggesting significant potential for discovering more myzostomid species. 

Phylogenetic analyses conducted in our study have unveiled discrepancies between certain phylogenetic placements and molecular data. Specifically, the COI sequences of *M. polycyclus* (DQ238202) and *M. toliarense* (DQ238201) exhibit a striking similarity, suggesting a potential misattribution, as previously highlighted by Summers and Rouse [2]. Furthermore, despite morphological alignment with the diagnostic features of *M. polycyclus*, our sequences differ significantly from those attributed to DQ238202. Therefore, we propose that the sequence likely belongs to *M. toliarense* rather than *M. polycyclus*.

Our research enhances the genomic dataset for myzostomids, refining both inter- and intraspecific genetic boundaries and diagnostic criteria. Notably, we identified substantial genetic distinctions between *M. scopus* sp. nov. and its closest relative, *M. susanae* (Figure 6, Figures S2 and S3). Moreover, our analyses revealed significant divergence among specimens of *M. ordinatum* sp. nov., scarcely distinguishable from *M. longitergum*, suggesting a potential species complex. Conversely, microscopic and genomic analyses of specimens identified as *M. ordinatum* sp. nov. unexpectedly revealed significant morphological variability (Figure 5), highlighting the necessity of an integrative taxonomic approach to prevent premature species delineations.

The correction of the COI sequence attribution for *M. polycyclus* underscores the importance of rigorous genetic verification in species identification. This is particularly pertinent in taxonomic groups characterized by limited diversity sampling, where erroneous assignments can pose challenges detectable only through comprehensive comparisons across multiple reference datasets. Our study thus contributes valuable insights into myzostomid biodiversity and underscores the ongoing need for genetic and morphological analyses to refine species boundaries accurately.

As noted previously by Summers and Rouse [2], myzostomids typically exhibit host specificity, with closely related species often parasitizing crinoids from the same evolutionary clade. The newly described species *M. scopus* sp. nov. and *M. ordinatum* sp. nov. were both discovered on *Comaster schlegelii*, a host also inhabited by myzostomids from related clades such as *M. susanae* and *M. longitergum,* consistent with prior findings. This supports the hypothesis of co-evolution between crinoids as hosts and myzostomids as highly specialized symbionts.

In contrast, certain myzostomid species demonstrate a broader host range across different taxa. For example, *M. polycyclus*, found on *C. parvicirrus* in our study, is also known to parasitize other feather stars of diverse genera and species (e.g., *Capillaster multiradiatus, Clarkcomanthus littoralis, Comanthus suavius*), suggesting it may be less host specific.

Moreover, our analysis underscores the considerable gap in knowledge regarding myzostomid biology. This limited understanding currently hinders assessments of their phylogenetic relationships, host specificity, and biogeographical distribution patterns.

## 5. Conclusions

The findings of this study illuminate the hidden biodiversity within marine macroinvertebrates, emphasizing the pivotal role of documenting these and numerous other symbiotic relationships, especially given ongoing environmental challenges [21,40]. It is noteworthy that many of these microscopic invertebrate symbionts can take on parasitic or vector roles, particularly within key habitat-forming taxonomic groups such as echinoderms, corals, and marine sponges. 

Our investigation underscores the urgent need for comprehensive, integrative research efforts that move beyond mere species enumeration. The complexity of marine ecosystems demands multidisciplinary approaches to untangle intricate relationships, providing crucial insights for informed decision-making in an increasingly dynamic global landscape.

## Figures and Tables

**Table 1:** Hosts and number of *Myzostoma* specimens studied.

Host	*Myzostoma polycyclus*	*Myzostoma solare* sp. nov.	*Myzostoma ordinatum* sp. nov.	*Myzostoma scopus* sp. nov.
*Comanthus parvicirrus*	18			
*Comanthus parvicirrus*	10			
*Comanthus parvicirrus*	5			
*Comaster schlegelii*		1	2	
*Comaster schlegelii*		6	25	
*Comaster schlegelii*		3	34	2
*Comaster schlegelii*			9	1

**Table 2:** Material deposited in public collections.

Species	Sampling Locality	Date of Sampling	Depth, m	Host	Voucher	GenBank Accession Number
*Myzostoma scopus* sp. nov.	18.20465; 109.16995	25 November 2009	5–8	*Comaster schlegelii*	ZMMU Pl-4886	OR864678
*Myzostoma solare* sp. nov.	18.20465; 109.16995	25 November 2009	5–8	*Comaster schlegelii*	ZMMU Pl-4887	OR864679
*Myzostoma ordinatum* sp. nov.	18.20465; 109.16995	25 November 2009	5–8	*Comaster schlegelii*	ZMMU Pl-4888	
*Myzostoma ordinatum* sp. nov.	18.20465; 109.16995	25 November 2009	5–8	*Comaster schlegelii*	ZMMU Pl-4889	OR864677
*Myzostoma ordinatum* sp. nov.	18.20465; 109.16995	25 November 2009	5–8	*Comaster schlegelii*	ZMMU Pl-4890	
*Myzostoma polycyclus*	18.20465; 109.16995	24 November 2009	3–5	*Comanthus parvicirrus*	ZMMU Pl-4891	OR864681
*Myzostoma polycyclus*	18.20465; 109.16995	24 November 2009	3–5	*Comanthus parvicirrus*	ZMMU Pl-4892	
*Myzostoma polycyclus*	18.20465; 109.16995	24 November 2009	3–5	*Comanthus parvicirrus*	ZMMU Pl-4893	OR864680

## Data Availability

Genetic data obtained in this study are available by Accession Numbers provided in Table 2.

## Supplementary Materials

The supplementary data can be accessed at the following https://www.mdpi.com/article/10.3390/ani14152265/s1. Table S1: Taxonomic names and GenBank accession numbers of partial COI sequences of Myzostomida used for molecular phylogenetic reconstruction. Figure S1: Ventral view of the anterior part of *Myzostoma ordinatum* sp. nov. with an everted introvert. Scale bar 200 µm (abbreviations: i—introvert, mc—marginal cirri, p—parapodia). Figure S2: Bayesian inference (BI) molecular phylogenetic tree of the genus *Myzostoma*, derived from partial COI sequences. Figure S3: Maximum likelihood (ML) molecular phylogenetic tree of the genus *Myzostoma*, derived from partial COI sequences. Figure S4: The distribution of myzostomids, as illustrated in Figure 6, spans across global marine biogeographical realms, following the framework established by Costello et al. [34] with modifications.
