# Supplementary material for: New Myzostomids (Annelida) in Symbiosis with Feather Stars in the Shallow Waters of the South China Sea (Hainan Island)
Source: Animals (Basel). 2024 Aug 4;14(15):2265. doi: 10.3390/ani14152265 (PMC11310986; doi:10.3390/ani14152265)
Supplement: Supplementary file 1 [file animals-14-02265-s001.zip › FigureS1_.pdf]

animals

MDPI

Article

New Myzostomids (Annelida) in Symbiosis with Feather Stars in the Shallow Waters of the South China Sea (Hainan Island)

Alexander Isaychev [1,†], Dimitry Schepetov [1,2,†], Yutong Zhou [1], Temir A. Britayev [3] and Viatcheslav N. Ivanenko [1,2,*]

[1] Biological Faculty, Shenzhen MSU-BIT University, Shenzhen 518172, China; isaychev1@yandex.ru (A.I.); d.m.schepetov@gmail.com (D.S.); 1120210251@smbu.edu.cn (Y.Z.)
[2] Department of Invertebrate Zoology, Lomonosov Moscow State University, Moscow 119992, Russia
[3] A.N. Severtsov Institute of Ecology and Evolution Russian Academy of Sciences, Moscow 129164, Russia; britayev@yandex.ru
* Correspondence: ivanenko.slava@gmail.com
† These authors contributed equally to this work.

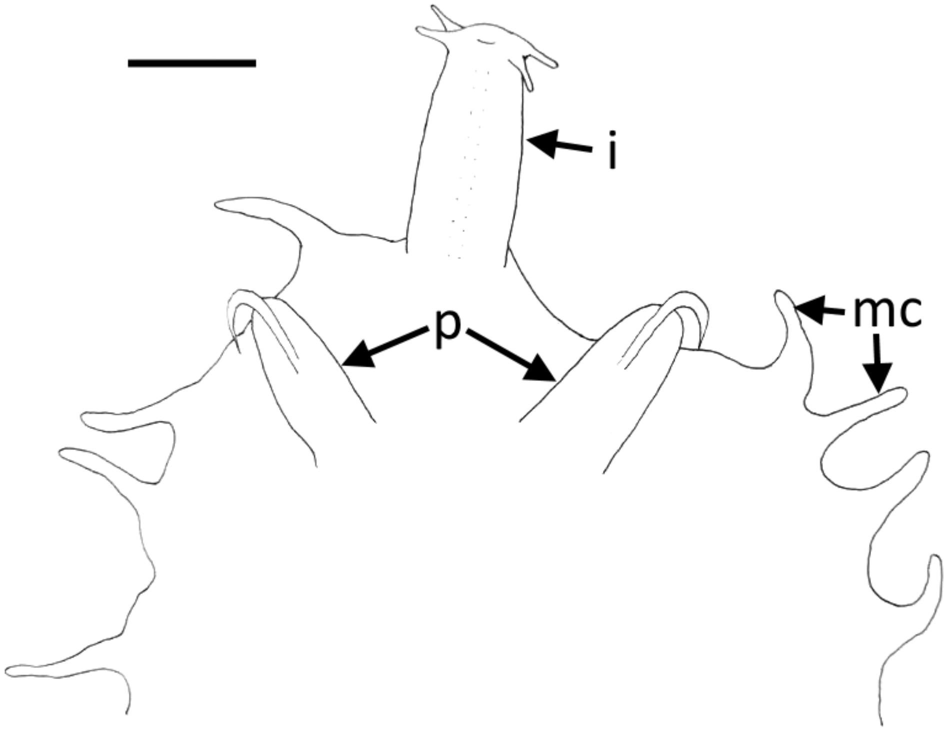

Figure S1: Ventral view of the anterior part of *Myzostoma ordinatum* sp. nov. with an everted introvert. Scale bar 200 µm (abbreviations: i – introvert, mc – marginal cirri, p – parapodia).

Citation: Isaychev, A.; Schepetov, D.; Zhou, Y.; Britayev, T.A.; Ivanenko, V.N. New Myzostomids (Annelida) in Symbiosis with Feather Stars in the Shallow Waters of the South China Sea (Hainan Island). *Animals* **2024**, *14*, 2265. https://doi.org/10.3390/ani14152265

Academic Editor: Jorg. D. Hardege

Received: 18 December 2023
Revised: 22 July 2024
Accepted: 29 July 2024
Published: 4 August 2024

Copyright: © 2024 by the authors. Submitted for possible open access publication under the terms and conditions of the Creative Commons Attribution (CC BY) license (https://creativecommons.org/licenses/by/4.0/).
