# Supplementary figures and images for: New Myzostomids (Annelida) in Symbiosis with Feather Stars in the Shallow Waters of the South China Sea (Hainan Island)
Source: Animals (Basel). 2024 Aug 4;14(15):2265. doi: 10.3390/ani14152265 (PMC11310986; doi:10.3390/ani14152265)

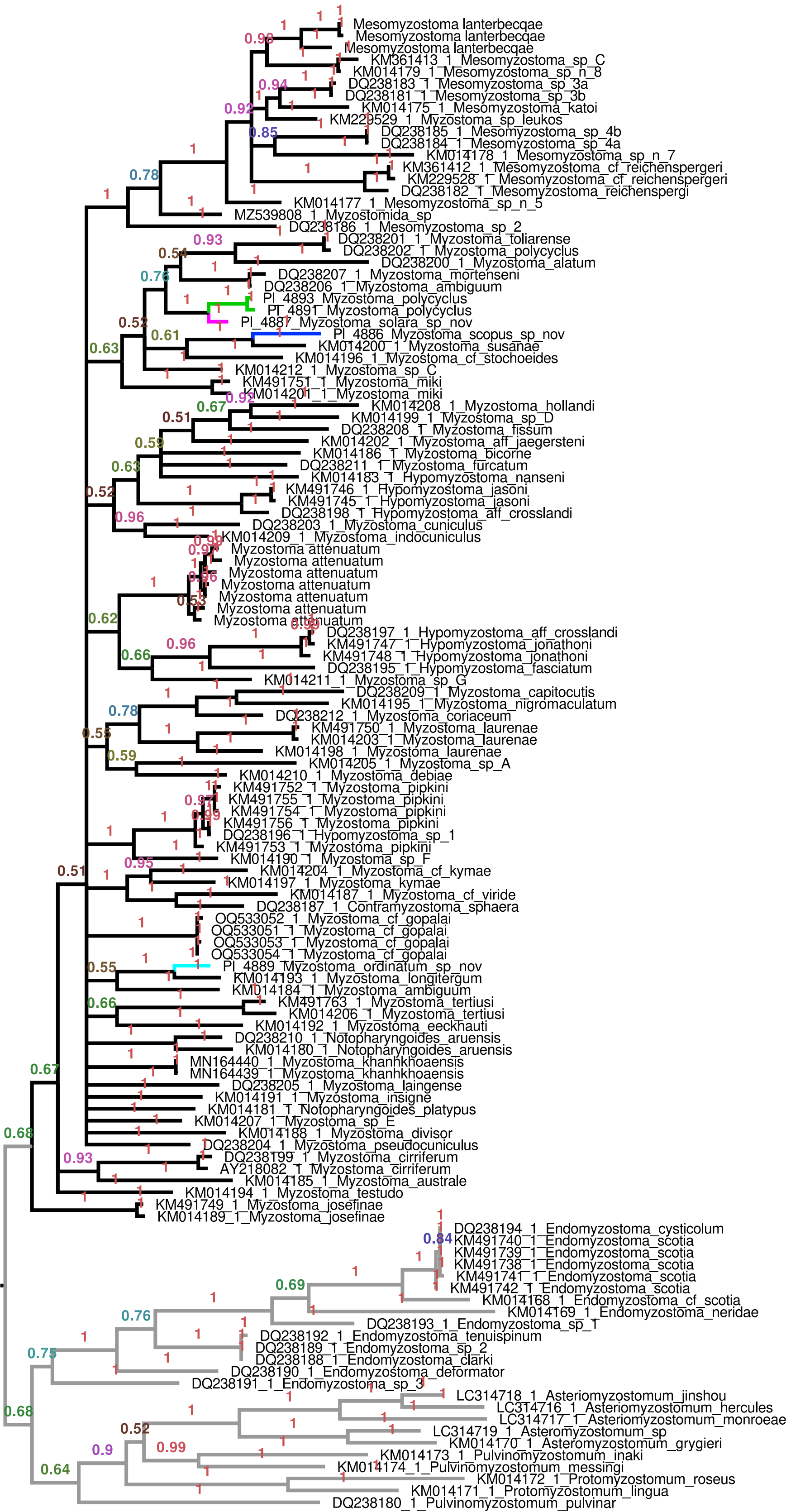

Supplement: Supplementary file 1 [file animals-14-02265-s001.zip › FigureS2.pdf]

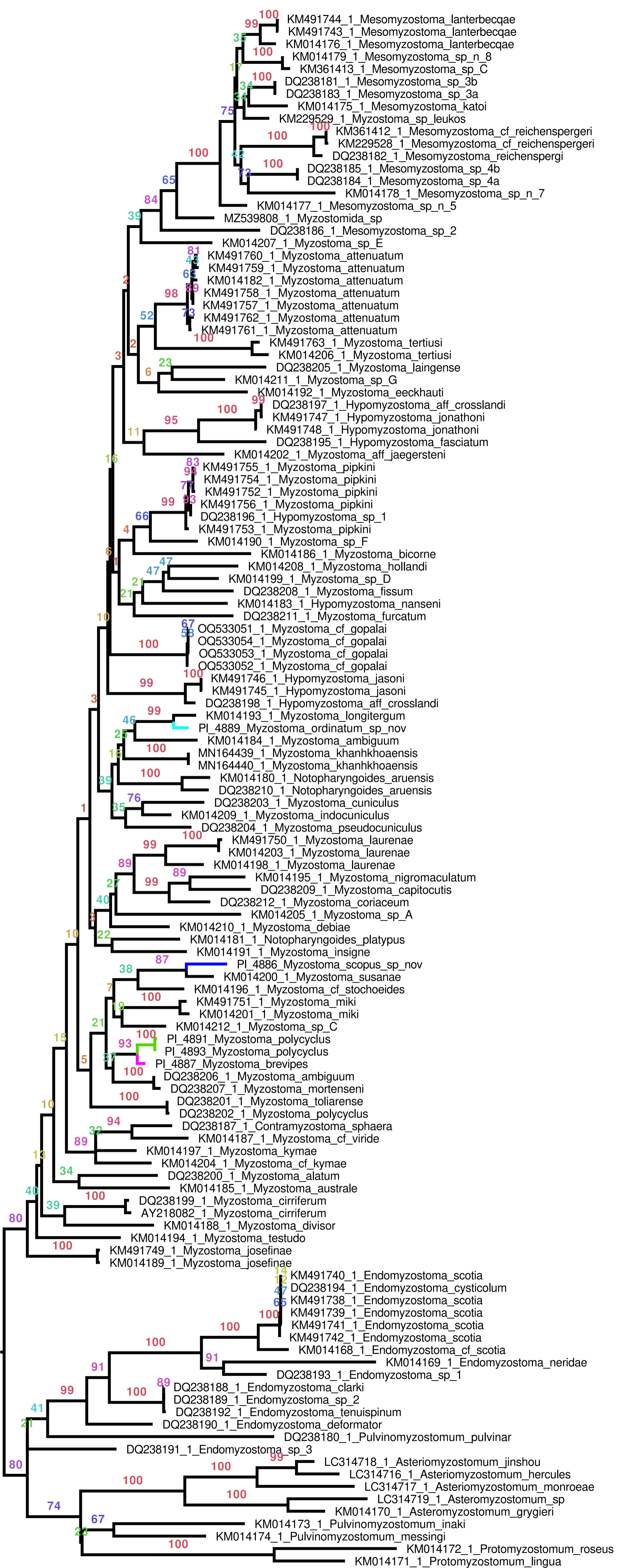

Supplement: Supplementary file 1 [file animals-14-02265-s001.zip › FigureS3_.pdf]
